# Impact of Clinical and Sociodemographic Factors on Quality of Life Following Coronary Artery Bypass Grafting: A Mixed-Methods Study

**DOI:** 10.7759/cureus.56781

**Published:** 2024-03-23

**Authors:** Abdulmajeed A Alzahrani, Abdullah K AlAssiri, Khalid E Al-Ebrahim, Zeyad T Ganbou, Meshal M Alsudais, Abdulmajeed M Khafagy

**Affiliations:** 1 Cardiac Surgery, King Abdulaziz University Hospital, Jeddah, SAU; 2 Cardiac Surgery, King Abdulaziz University, Faculty of Medicine, Jeddah, SAU

**Keywords:** ischemic heart disease, health-related quality of life, coronary artery disease, coronary artery bypass grafting, quality of life

## Abstract

Introduction: Coronary artery bypass grafting (CABG) is an essential surgical management modality for patients with coronary artery disease. Health-related quality of life (HRQoL) has become important because of the significant decrease in the mortality rate associated with CABG. We aimed to explore the factors that affect the quality of life after CABG.

Methods: This study used a descriptive correlational design to assess the determinants of HRQoL using the 36-item Short Form Health Survey questionnaire (SF-36). Patients who underwent CABG at King Abdulaziz University Hospital (KAUH), Jeddah, Saudi Arabia, between March 2015 and December 2021 were enrolled in this study. Overall, 275 participants were eligible for our study, of which 84 were found to be valid for analysis. Phone contacts were made directly with the patient after briefly explaining the study. Scores and clinical data were investigated using multivariable linear regression analysis.

Results: Subscales of role limitations due to physical issues had the lowest mean scores, followed by vitality and general health (57.4 ± 44.7; 60.4 ± 25.6; 64.1 ± 22.6), respectively. However, social functioning (78.9 ± 29.0) and pain (75.1 ± 29.9) had the highest scores of all subscales. A history of congestive heart failure (CHF) was independently associated with lower scores for physical role limitations (p = 0.021), vitality (p = 0.001), general health (p< 0.001), and mental health (p = 0.011). Lower mental health scores were also predicted by being a widow (p = 0.030), whereas lower general health scores were predicted by being unemployed (p = 0.001) and having a peripheral vascular disease (PVD) (p = 0.043). Additionally, the development of postoperative complications was an independent predictor of lower physical functioning (p = 0.028) and vitality (p = 0.043). Regarding the number of grafts, cardiopulmonary bypass, and cross-clamp time, no significant impact was found on any of the SF-36 subscales (p> 0.05).

Conclusion: The postoperative decline in HRQoL was attributed to comorbidities such as CHF and PVD, postoperative complications including bleeding and wound infection, as well as unemployment and widowed status. Therefore, choosing the appropriate patients for surgery and post-discharge follow-up may enhance HRQoL.

## Introduction

Cardiovascular disease (CVD) is a group of diseases that affect the functioning of the heart and blood vessels. According to the World Health Organization (WHO), CVD and disability are the main causes of death in developed countries. In 2013, it contributed to ˃17.3 million deaths; by 2030, this number could increase to ˃23.6 million [1]. According to the Ministry of Health Statistical Yearbook published in 2016, 7,740 deaths in Saudi Arabia were attributed to circulatory system defects. This increase in CVD directly impacts the death rate [2]. Government cardiac facilities are becoming more common in Saudi Arabia, which has one of the most evolving healthcare systems. Overall, 16,013 cardiac, thoracic, and vascular procedures were performed in the country in 2021, according to the Saudi Ministry of Health [2]. However, post-cardiac surgery health-related quality of life (HRQoL) has received little attention in Saudi studies.

Coronary artery bypass grafting (CABG) is considered an essential treatment and is commonly used worldwide for patients with coronary artery disease [3]. Coronary artery bypass grafting has significantly improved the therapeutic outcomes for ischemic heart disease (IHD) for more than 25 years [4]. Coronary artery bypass grafting also improves the survival of patients with multivessel coronary disease by reducing damage to the coronary arteries. The operation involves shunting blood around an obstruction in the coronary arteries, which provides the heart with blood and oxygen [5]. The usual assessment of CABG impact has focused on objective measures, such as morbidity, mortality, and clinical dysfunction, to assess treatment goals. Since there has been a significant decrease in the operative mortality rate with CABG over the past 30 years, quality of life (QoL) has become important to assess the advantages and risks of CABG and help patients make better choices [6].

Quality of life relates to the patient's perception of changes in their health status following a surgical procedure. It signifies the degree to which an individual experiences a state of well-being, comfort, and the capacity to participate in regular activities [7]. It is evaluated as learning more about a person’s health benefits. This allows patients to determine the quality of care by predicting what values will be associated with different outcomes. Regretfully, there is not a sufficient improvement in all areas of patients' QoL following CABG, and some even report worse HRQoL [8]. Following CABG, patients frequently experience pain, discomfort, depressive symptoms, impatience, a loss of overall well-being, and an inability to return to their preoperative level of functioning. These emotions may significantly lower the patient's QoL [9]. Changes in HRQoL after CABG are correlated with pharmacological therapy, non-medical variables, and numerous other factors, including sex, diabetes mellitus, obesity, low ejection fraction, and smoking. Therefore, knowing the factors affecting HRQoL after CABG can help with prognosis and reduce or eliminate modifiable factors. Physical, mental, and social aspects are the three most crucial components of QOL in CABG [10]. These measurements provide the patient’s perception of their ability to identify the factors most responsible for their overall HRQoL [11].

Studying and evaluating QoL issues is crucial because it predicts an individual’s ability to deal with their illness and improve their health and well-being, particularly after undergoing major surgeries such as CABG. Furthermore, because of the significant annual increase in CABG cases, evaluating HRQoL is crucial to providing better care and control of the disease and condition. There is a lack of large numbers of descriptive studies about QoL after CABG in our Arabian Gulf region using reliable, validated instruments. Therefore, this study aimed to assess post-CABG HRQoL using the 36-item Short Form Health Survey questionnaire (SF-36) and its influencing variables, including sociodemographic factors, comorbidities, and operative variables such as the number of grafts, duration of cardiopulmonary bypass, and cross-clamp time.

## Materials and methods

Study design and setting

This cross-sectional, questionnaire-based, non-randomized study was conducted in the cardiac surgery division of the surgery department at King Abdulaziz University Hospital (KAUH) in Jeddah, Saudi Arabia, from September 2022 to December 2022 and was approved by the institutional ethical committee (approval number: 438-22).

All participants were notified of the study objectives and response confidentiality, and verbal consent was obtained from all participants during the call after explaining the aim and benefits of the study. All clinical data retrieved were anonymized, ensuring patients' confidentiality. All patients who underwent isolated CABG between March 2015 and December 2021 at KAUH were invited to participate in the study. Surgical procedures were conducted using classic cardiopulmonary bypass techniques and myocardial protection by crystalloid cardioplegia with or without topical cooling. No off-pump or minimally invasive technique was used in the study population. The grafts used during the surgery were internal mammary artery and vein grafts. Patients who had undergone any prior cardiac, thoracic, aortic, or vascular surgery other than CABG were excluded from the study.

**Figure 1:**
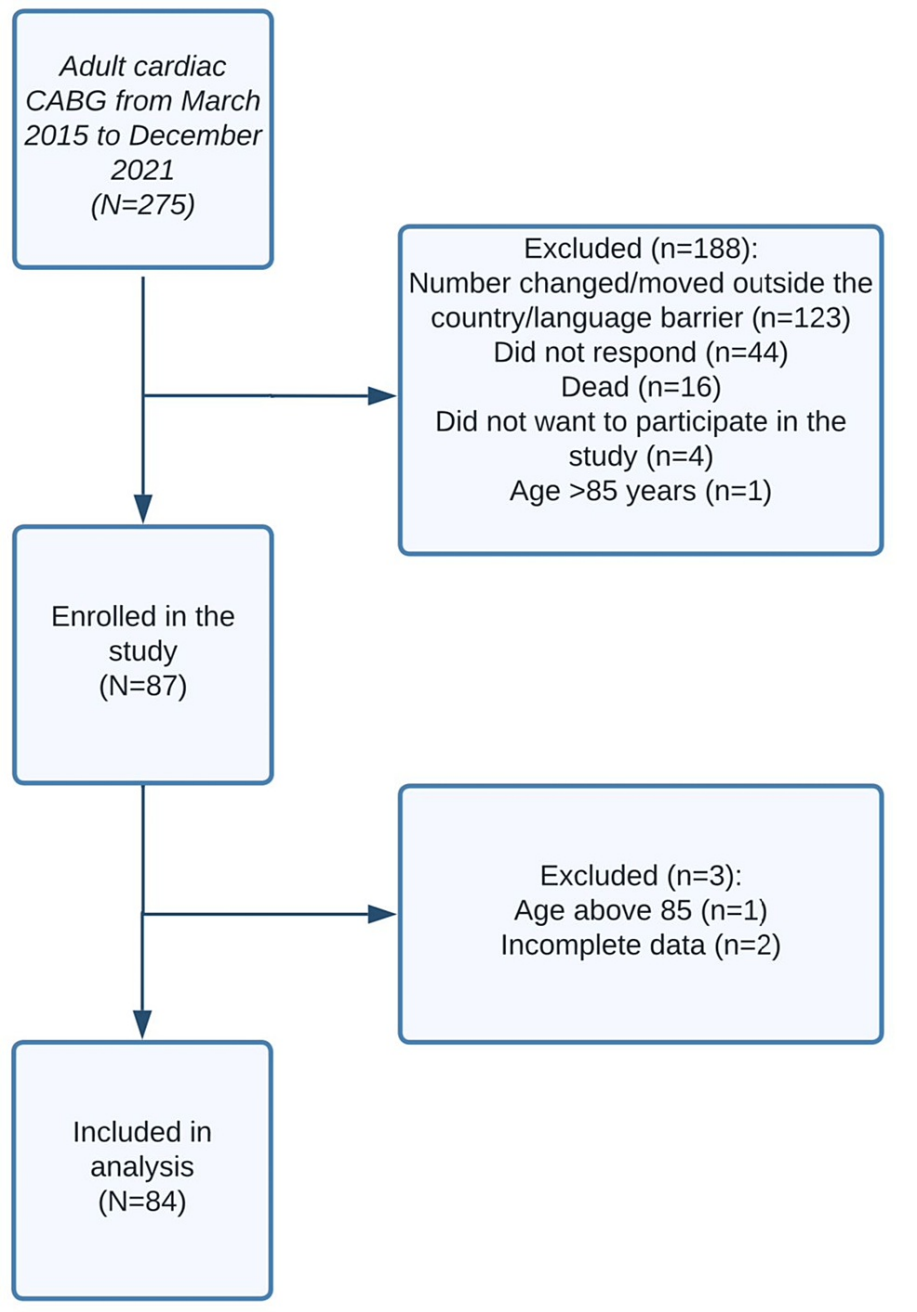
Flowchart showcasing included and excluded patients CABG: coronary artery bypass grafting

Overall, 275 patients who met the inclusion criteria, including those who underwent primary isolated CABG, were enrolled. In total, 188 patients were excluded. One hundred and twenty-three patients were excluded due to being located outside the country, changes in their contact information, or language barriers. Additionally, 44 patients were excluded due to non-responsiveness, 16 patients were excluded as they were deceased, four patients were excluded due to their refusal to participate in the study, and one patient was excluded due to being over the age of 85 (Figure 1). The remaining 87 patients were invited to participate in the study via telephone calls using their numbers registered in our hospital system, and phone contact was made directly with the patient. Subsequently, patients’ medical records in the cardiac surgery database were retrospectively reviewed, and they were interviewed via telephone to answer the SF-36 questionnaire. Verbal informed consent was obtained after a brief explanation of the study objectives and design.

Surgical technique

In the surgical technique for CABG, an open median sternotomy was performed. This involved inducing hypothermic cardiopulmonary bypass using aortic cannulation. All patients underwent surgery on a cardioplegically arrested heart with cold blood cardioplegia. Distal bypasses were initiated, prioritizing the internal mammary artery for grafting to the left anterior descending coronary artery, and reversed saphenous veins were used for grafting to other coronary arteries. The procedure included rewarming, weaning from bypass, achieving hemostasis, and routine closure.

Data collection process

This study included two sections. The first was a retrospective review of patient records by entering a specific medical record number for each patient. Clinical data were obtained from the registry database, medical and outpatient notes, intensive care unit (ICU) charts, operation reports, and coronary care unit notes, including sociodemographic data, comorbidities such as diabetes mellitus and dyslipidemia, postoperative complications such as wound infection and bleeding, history of both arrhythmia and stroke pre- and post-CABG, and cardiac surgery-related data such as the number of grafts, duration of cardiopulmonary time (CBP), and cross-clamp time (ACX). All the required medical records and data were documented in the medical-hospital system, and Google Sheets and Google Forms (Google Inc., Mountainview, CA) were used for data collection. Each data collector was only able to complete the questionnaire once, using their email addresses as a unique identifier, and all responses were uploaded to a Microsoft Excel (Microsoft Corp., Redmond, WA) file that could only be accessed by researchers. The data collection was done separately for each patient by using the medical record number, and the Google Forms announced to the data collector if there were any missing or incomplete data to ensure the completeness and accuracy of the data. The records were retrospectively analyzed when patients consented to participate in the study during the phone calls. 

The second part was the administration of an SF-36 questionnaire to collect data regarding HRQoL using a valid and reliable Arabic version of the SF-36 survey with an acceptable level of homogeneity across all items since it is a universal questionnaire for evaluating the effect of surgical and medical treatment on HRQoL [12]. The SF-36 questionnaire is widely used in medical practice and research and has been applied to patients undergoing cardiac surgery; it is sufficiently sensitive to identify any changes in pain, function, and overall health [13]. The SF-36 is a 36-item questionnaire that measures eight multi-item dimensions of health as follows: physical functioning (10 items), social functioning (two items), role limitations due to physical problems (four items), role limitations due to emotional problems (three items), mental health (five items), energy/vitality (four items), pain (two items), and general health perception (five items). For each dimension, the item scores were coded, summed, and transformed on a scale from 0 (the worst possible health status measured using the questionnaire) to 100 (the best possible health status). Additionally, two standardized summary scores were calculated from the SF-36 as follows: physical and mental health component summaries.

Definitions

A healthy weight was defined as 18.5-24.9 kg/m^2^, overweight at 25-29.9 kg/m^2^, and obesity at ≥30 kg/m^2^. The left ventricular ejection fraction (LVEF) was also considered normal, mild, moderate, and severe dysfunctions at 50%-70%, 40%-49%, 30%-39%, and <30%, respectively.

Scoring

The SF-36 survey items were recorded and averaged based on the formal guidelines of the RAND Medical Outcomes Study [14]. The 36 items were categorized into eight subscales as follows: physical functioning, physical role limitations, emotional role limitations, energy, emotional well-being, social functioning, pain, and general health. The scores for each subscale ranged from 0 to 100.

Statistical analysis

Microsoft Excel was used for data entry, and statistical analysis was performed using RStudio version 4.1.1 (The R Core Team, R Foundation for Statistical Computing, Vienna, Austria). A statistician worked on the research's statistical analysis. Statistical software packages, including IBM SPSS Software, Stata, and others, have advantages and disadvantages. The statistician used RStudio because it is comprehensive, it is easier to manage the code on it, and the statistician is more accustomed to it. Any missing or duplicated data, if available, were handled by deleting them to ensure all data were accurate. Categorial variables were chosen depending on whether they take on an infinite set of values. While categorical variables take on a limited set of fixed values, confounders were controlled using regression analysis. The internal consistency of the different subscales was investigated using Cronbach’s alpha coefficient. Categorical and continuous data were expressed as frequencies and percentages and median and interquartile range (IQR) or mean ± standard deviation (SD), respectively. Factors associated with each subscale of QoL were assessed by constructing several multiple linear regression models, where the score of each subscale and the sociodemographic, clinical, and operative variables were used as dependent and independent variables, respectively. Significantly associated predictors were identified based on a stepwise forward selection method, and they were reported exclusively along with their adjusted beta coefficients and 95% confidence intervals (95% CIs). Statistical significance was set at p<0.05.

## Results

Sociodemographic characteristics

Initially, data were collected from 87 patients. However, we excluded data from one patient aged 85 and two without available data relevant to sociodemographic characteristics. Therefore, data from 84 patients were analyzed in this study. Less than half of the patients were aged between 50 and 60 (45.2%), with overweight patients comprising 37 (44.0%). Most patients were male (71, 84.5%), married (74, 88.1%), and 51 (60.7%) of the study sample were unemployed. Based on smoking status, current and former smokers constituted seven (8.3%) and 43 (51.2%) participants, respectively (Table 1).

**Table 1:** Sociodemographic characteristics BMI: body mass index

Parameter	Category	N (%)
Age (in years)	<50	10 (11.9%)
	50–60	38 (45.2%)
	>60	36 (42.9%)
Sex	Male	71 (84.5%)
	Female	13 (15.5%)
BMI	Healthy	25 (29.8%)
	Overweight	37 (44.0%)
	Obese	22 (26.2%)
Marital status	Single	1 (1.2%)
	Married	74 (88.1%)
	Divorced	7 (8.3%)
	Widow	2 (2.4%)
Employment status	Employed	33 (39.3%)
	Unemployed	51 (60.7%)
Smoking history	Never smoked	34 (40.5%)
	Current smoker	7 (8.3%)
	Former smoker	43 (51.2%)

Clinical, operative, and postoperative characteristics

**Figure 2:**
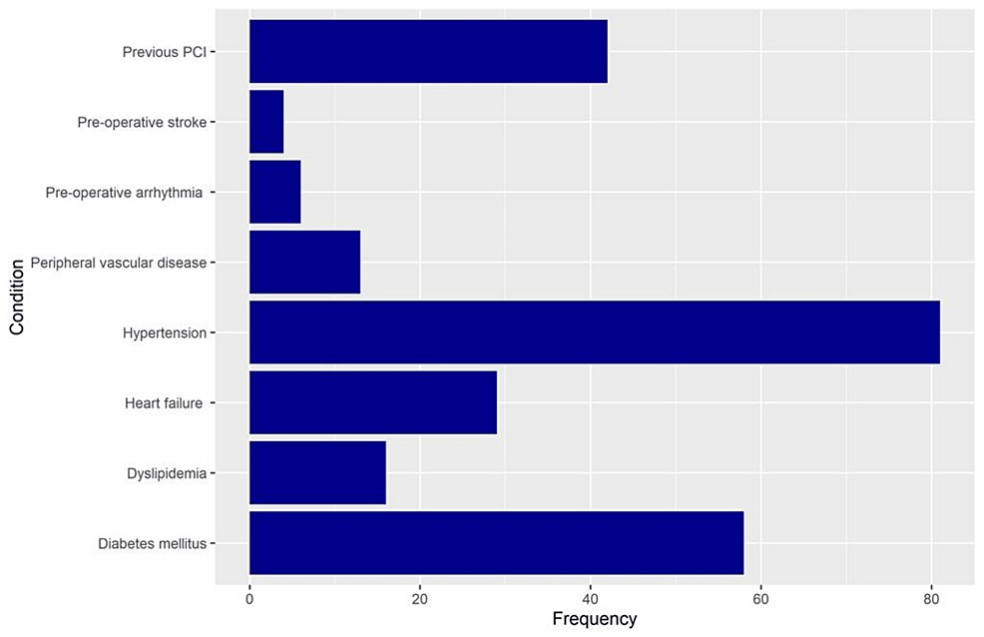
Frequency of comorbidities among the patients under study PCI: percutaneous coronary intervention

Half the patients (50.0%) had previously undergone percutaneous coronary interventions. The most common chronic conditions among the patients were hypertension (80, 96.4%), diabetes mellitus (57.96, 69.0%), and heart failure (28, 34.5%) (Figure 2). The median (IQR) pre-operative LVEF was 50.0% (range: 40.0%-55.0%). Considering the operative characteristics, the median (IQR) cardiopulmonary time was 114.5 minutes (range: 91.0-128.5 minutes), and the cross-clamp time was 67.5 minutes (range: 55.0-87.0 minutes). Postoperatively, the median (IQR) length of hospital stay was 12.0 days (range: 8.0-14.5 days). Postoperative complications occurred in 11 patients (13.1%), among whom bleeding occurred in eight (72.7%). Table 2 presents more details regarding the operative and postoperative characteristics.

**Table 2:** Clinical, operative, and postoperative characteristics *The variable has six missing values; ¥ The variable has one missing value; ‡ The variable has four missing values LVEF: left ventricular ejection fraction

Parameter	Category	N (%)	p-value
LVEF*	Normal	42 (53.8%)	>0.05
	Mild dysfunction	26 (33.3%)	>0.05
	Moderate dysfunction	7 (9.0%)	>0.05
	Severe dysfunction	3 (3.8%)	>0.05
Number of grafts^¥^	1–3	49 (59.0%)	>0.05
	≥4	34 (41.0%)	>0.05
Duration of cardiopulmonary time (minutes)	<90	19 (22.6%)	>0.05
	90 to <120	29 (34.5%)	>0.05
	≥120	36 (42.9%)	>0.05
Cross-clamp time (minutes)	<60	24 (28.6%)	>0.05
	60 to <90	43 (51.2%)	>0.05
	≥90	17 (20.2%)	>0.05
Length of hospital stay (days)^¥^	<10	30 (36.1%)	>0.05
	10–20	45 (54.2%)	>0.05
	>20	8 (9.6%)	>0.05
Postoperative complications	Yes	11 (13.1%)	>0.05
If yes, what type of complication	Bleeding	8 (72.7%)	>0.05
	Stroke	1 (9.1%)	>0.05
	shock	1 (9.1%)	>0.05
	Wound infection	1 (9.1%)	>0.05
Known case of postoperative arrhythmia^‡ ^	Yes	10 (12.5%)	>0.05

Description of the SF-36 scales

**Figure 3:**
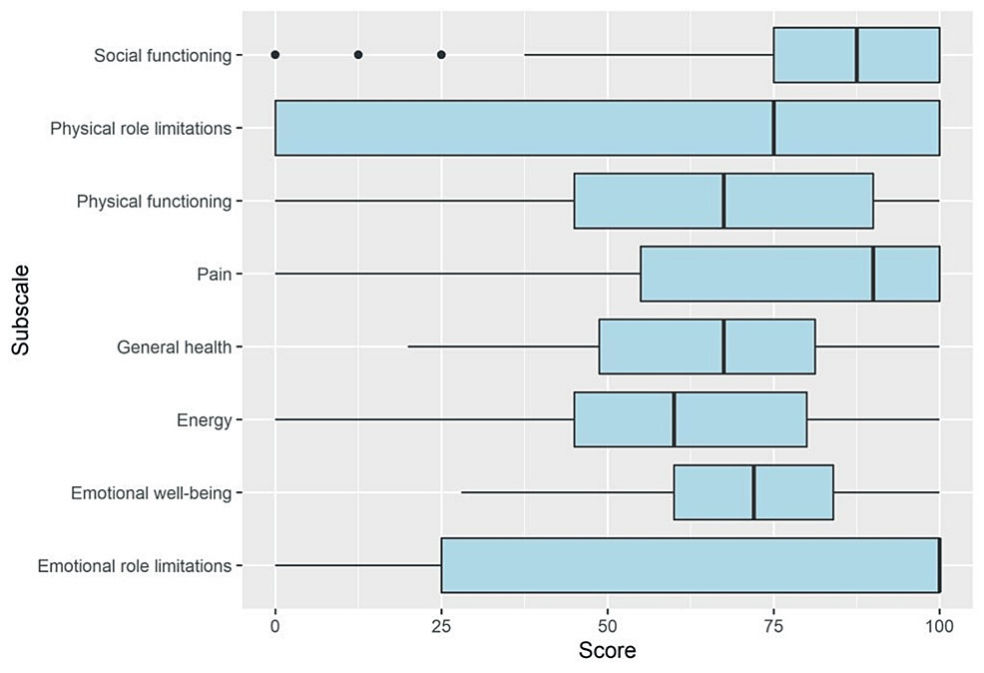
Boxplots depicting the descriptive numerical statistics of different SF-36 survey subscales

The SF-36 questionnaire is categorized into eight variables/domains: physical functioning, physical role, emotional role, vitality, mental health, social functioning, pain, and general health. Table 2 lists the mean and SD of the domains. The variables with relatively higher means were social functioning (78.9 ± 29.0), pain (75.1 ± 29.9), and mental health (71.7 ± 17.0). In comparison, the lowest scores were observed in role limitations due to physical problems (57.4 ± 44.7), vitality (60.4 ± 25.6), and general health (64.1 ± 22.6). Table 3 and Figure 3 present detailed descriptive data for the different SF-36 subscales.

**Table 3:** Descriptive and reliability analyses of the different SF-36 subscales SD: standard deviation; IQR: interquartile range; HRQoL: health-related quality of life; SF-36: 36-item Short Form Health Survey

Characteristic	Items	Mean ± SD	Median (IQR)	Min-Max
Physical functioning	10	65.7 ± 27.2	67.5 (45.0, 90.0)	0.0–100.0
Role limitations due to physical problems	4	57.4 ± 44.7	75.0 (0.0, 100.0)	0.0–100.0
Role limitations due to emotional problems	3	67.5 ± 43.0	100.0 (25.0, 100.0)	0.0–100.0
Energy/Vitality	4	60.4 ± 25.6	60.0 (45.0, 80.0)	0.0–100.0
Mental health/Emotional well-being	5	71.7 ± 17.0	72.0 (60.0, 84.0)	28.0–100.0
Social functioning	2	78.9 ± 29.0	87.5 (75.0, 100.0)	0.0–100.0
Pain	2	75.1 ± 29.9	90.0 (55.0, 100.0)	0.0–100.0
General health	5	64.1 ± 22.6	67.5 (48.8, 81.2)	20.0–100.0
Overall HRQoL	36	66.4 ± 21.9	70.8 (47.6, 85.8)	15.8–97.2

Predictors of the health-related quality of life

Based on the multiple linear regression analysis results, a history of heart failure was independently associated with lower scores of physical role limitations (beta = -36.5, 95% CI, -67.1 to -5.92, p = 0.021), energy (beta = -27.7, 95% CI, -43.7 to -11.7, p =0.001), general health (beta = -26.7, 95% CI, -41.6 to -11.9, p < 0.001), and emotional well-being (beta = -13.5, 95% CI, -23.8 to -3.29, p = 0.011). Lower scores of emotional well-being were also predicted by being a widow (beta = -60.2, 95% CI, -90.5 to -6.01, p = 0.030), whereas those of general health were predicted by being unemployed (beta = -27.3, 95% CI, -43.0 to -11.5, p = 0.001) and having a peripheral vascular disease (PVD) (beta =-21.0, 95% CI, -41.3 to -0.66, p = 0.043). Additionally, the development of postoperative complications was an independent predictor of lower physical functioning (beta =-26.3, 95% CI,-49.6 to-3.00, p = 0.028) and energy (beta =-24.0, 95% CI,-47.2 to-0.75, p =0.043) (Table 4).

**Table 4:** Results of the multiple linear regression models for the health-related quality of life subscales (the SF-36 questionnaire) *All variables in the model were non-significant (NS); ¥Only variables with significant p-values are reported CI: confidence interval; SF-36: 36-item Short Form Health Survey

Dependent variable	Independent variable(s)^¥^	Standardized beta (95% CI)	p-value	R-square
Physical functioning	Postoperative complications	-26.3 (-49.6 to -3.00)	0.028	35.8
Role limitations due to physical problems	Heart failure	-36.5 (-67.1 to -5.92)	0.021	17.4
Role limitations due to emotional problems*	NS	NS	NS	15.7
Energy/Vitality	Heart failure	-27.7 (-43.7 to -11.7)	0.001	26.8
	Postoperative complications	-24.0 (-47.2 to -0.75)	0.043	
Mental health/Emotional well-being	Widow	-60.2 (-90.5 to -6.01)	0.030	28.7
	Heart failure	-13.5 (-23.8 to -3.29)	0.011	
Social functioning*	NS	NS	NS	11.8
Pain*	NS	NS	NS	6.8
General health perception	Unemployed	-27.3 (-43.0 to -11.5)	0.001	29.8
	Peripheral vascular disease	-21.0 (-41.3 to -0.66)	0.043	
	Heart failure	-26.7 (-41.6 to -11.9)	<0.001	

## Discussion

This is a cross-sectional, questionnaire-based study planned in a descriptive correlational design to assess the determinants of HRQoL in the minimum postoperative period one year after primary isolated CABG via classic open cardiac surgery. Quality of life is a multidimensional assessment of personal perception of the physical and psychosocial aspects influenced by the disease and its medical or surgical intervention. However, because many instruments apply various measurement definitions, what exactly defines HRQoL remains unclear [15]. Quality of life, as assessed by the SF-36 and its elements, is an expression of the way patients react and perceive their health status and the non-medical implications of their daily lives [16]. Despite a CABG operation improving IHD symptoms, this is not always reflected in an improvement in quality of life after surgery. Overall physical and mental health status as well as HRQoL are affected by the operation; one of the main predictors of a patient's medical prognosis is the patient's perspective and level of satisfaction. Therefore, we specifically selected the SF-36 health questionnaire, which contains subjective evaluations of HRQoL rather than physicians’ perceptions.

Here, most patients who underwent primary isolated CABG were male, which is consistent with the literature and an expected result because males are at a greater risk of experiencing coronary artery disease and undergoing CABG surgery [17]. However, other studies have shown that the average CABG mortality rate in women is higher than that in males. The size of the coronary arteries, baseline variations in clinical risk factors, and unfavorable cardiovascular profiles found in women all contribute to sex disparities in acute outcomes [18]. Even after assessing pre-existing risk factors, women's recovery after one year is not as fast as men's. Women are more likely to experience subjective cognitive issues, higher anxiety, a decline in abilities for daily living, a reduction in work-related activities, and a decrease in their ability to exercise. Women, therefore, have a greater probability than men to be using cardiac prescription medications, have a more bedridden and sedentary lifestyle, experience poorer health, have multiple coronary risk factors that continue to exist after CABG, and experience less relief of angina and dyspnea [19]. Occasionally, this disparity was minimized after adjusting for internal mammary artery graft use, age, and body surface area [20]. Consequently, knowing the reasons for sex differences in outcomes after CABG can optimize HRQoL.

The mean SF-36 subscale scores ranged from 57 to 78. This finding is similar to another study, which revealed that subscale scores ranged from 59 to 79 [17]. This study showed improvements in social functioning, pain, and mental health. Interestingly, despite these improvements, the patient's general health perception and overall reported HRQoL were negatively affected. Additionally, of the SF-36 subscales, the physical functioning and role limitation due to physical problems subscale showed declined scores, which was also observed in another recent cross-sectional study [21-24]. A possible explanation is that a high percentage of our study population was aged ˃60 years; perhaps having undergone major surgery, such as CABG, impacted their general health combined with the aging process, particularly for older patients, which may also account for this leveling effect on general health perception [18].

Older age is frequently associated with reduced HRQoL [25]. MacDonald et al. reviewed 200 patients ˃75 years who underwent CABG and were examined using the SF-36 Health Survey, in which they observed a higher rate of postoperative complications leading to death and disability in this group than in younger patients [26]. Previous studies have proven the impact of age as the most important factor because the HRQoL of older patients after cardiac surgery was similar to that of an age-matched general population. The risk factors of comorbidities such as hypertension, diabetes, and chronic obstructive pulmonary disease are anticipated to trigger operative mortality since they're highly common in this patient's age group [23]. The risk of surgical mortality is greatly increased by postoperative renal failure and respiratory failure. Prevention measures such as early extubation, head of the bed elevation, early mobilization, and aspiration protective measures should be addressed in these very elderly patients in order to limit respiratory problems [24]. According to a study recommendation, older people require interventions such as cardiac rehabilitation programs to lower morbidity, increase functionality, and improve QoL [18].

Congestive heart failure (CHF) in this study was diagnosed clinically either by the referring cardiologist or the cardiac surgeon, and the left ventricular systolic function was assessed through an echocardiography examination. In a cross-sectional study of patients with CHF who were under consideration for heart transplantation, the physical and mental component scores of the SF-36 were correlated with the severity of CHF. Thus, these mental components are more adversely affected [27]. However, our study showed that CHF predicted low mental score components and is involved in general health perception, vitality, and role limitations due to physical problems. A more in-depth investigation into the HRQoL of this specific group of patients showed that some cross-linking and collaboration exists between the mental and physical elements of the QoL in such patients [27]. The physical symptoms in these patients can make them more depressed and can cause a rapid deterioration in their physical and mental HRQoL [28]. Although the results of this study require validation, they may be used to identify patients with heart failure who are at risk of having a lower HRQoL after the operation [29].

The results of our study showed a significant negative impact on general health perceptions related to PVD, which is defined as chronic ischemia of the lower limbs. It is not surprising that a comparative study found that the impact of PVD on most SF-36 domains of HRQoL was more prominent than that of IHD [30]. Variations in the results between the two patient groups may have been masked if the patient had both coronary heart disease and PVD. The high rate of PVD in patients with coronary heart disease might confound a comparison [28].

Postoperative complications such as bleeding, stroke, shock, and wound infection had a significant negative impact on both physical functioning and vitality among patients undergoing CABG. Additionally, the mental component of HRQoL is significantly affected by infectious diseases such as septicemia, sternal wound infections, and leg vein harvest wound infections after CABG [13]. Furthermore, a large community-based study of many subgroups of surgical patients after hospital discharge, including those receiving CABG, showed that surgical site wound infections diagnosed after discharge had the same negative impact on self-reported HRQoL [31]. A cross-sectional study evaluated 208 patients who underwent elective CABG and found that postoperative complications are an independent predictor of poor QoL six months after surgery. Postoperative complications are associated with more frequent hospitalizations, longer postoperative hospital stays, and illnesses that affect the patients’ HRQoL [32]. A small exploratory study showed that adding a home-based rehabilitation program for older patients undergoing CABG or valve surgery may enhance their functional status and decrease the duration of hospitalization [33]. Patients who have had CABG recover more quickly when they receive pulmonary and cardiac rehabilitation programs, and they may have better heart function and quality of life after cardiac rehabilitation [34, 35]. Therefore, avoiding postoperative complications is crucial for a better quality of life since these complications are nightmares and frightening to patients and may risk their lives. Furthermore, strict surgical protocols and specific guidelines must be followed to minimize the occurrence of complications such as bleeding, sternal wound infection, acute kidney injury, diaphragmatic paralysis, and pleural effusion, requiring multiple unexpected emergency room readmissions. Moreover, the future of cardiac surgery is rapidly changing, and interventional surgery is promising for improving postoperative QoL [36, 37].

The patients in our study reported a relatively high pain score, which indicated a significant decrease in bodily pain. The reported reduction in physical pain following CABG may be attributed to the resolution of angina. However, another study showed that 21% of patients stated that they were still experiencing moderate-to-severe pain after 12 months, and 61% indicated they were still feeling pain. The same study found that approximately 25% of the participants still had angina pain. At 12 months, there was an unexpectedly high prevalence of severe-to-very severe leg/arm wound pain and sternotomy wound pain in 12.8% and 17% of patients, respectively. This severely impacted HRQoL 12 months postoperatively, making these patients more likely to have poor HRQoL [37]. Therefore, patients should be aware of the expected period in which their pain should be resolved; however, those who do not achieve a satisfactory level of pain relief should be evaluated for further management.

A significant finding of this study was that a high percentage of patients who underwent isolated CABG were unemployed. Additionally, a lower general health score was strongly associated with unemployment, which was statistically significant. Better functional status, employment, and involvement in sports are consistently linked to a higher QoL [38]. These results indicate that older people may have adapted to chronic heart disease over a longer period since they are more likely to be unemployed or have a sedentary lifestyle [39]. However, another study showed that half of the patients returned to work within approximately three months, and 80% returned within a year after CABG surgery. The longer the individual had been off work preoperatively, the longer it took them to return to work. Between 50% and 85% of patients returned to their old jobs, depending on how long they had been off work before surgery. Although the percentages were lower, a favorable trend for full-time and part-time employment was observed for women [40].

Widowed marital status has a significant negative impact on the mental component of HRQoL. A randomized controlled trial showed that widowed women had significantly lower HRQoL total scores than men at three, six, and 12 months postoperatively [38]. Close personal relationships are associated with better health outcomes due to psychological and material resources that help people overcome stress, and marital living status has been used as a surrogate for social support [41, 42]. Furthermore, another study conducted at a major medical center for patients undergoing cardiac surgery indicated that marriage had a significant protective impact on survival for approximately five years after heart surgery. Additionally, unmarried male or female patients undergoing heart surgery have an overall mortality risk of almost twice as high. Unmarried people had a 233% higher chance of dying within the first three months postoperatively and a 71% higher chance of dying within the next five years than married people [43]. Particularly, women recovered from cardiac surgery slower than men, and their HRQoL was lower [41]. This suggests that women, particularly those who live alone, require follow-up care and support to manage their symptoms and recover more quickly. Individuals at risk may benefit from individualized interventions that include evaluation of modifiable comorbidities such as depression, illness-related disability, and previous psychological stress that need more social support [41]. Therefore, including a separate assessment for social support would likely provide a more comprehensive evaluation of the association between marital status, social support, and HRQoL, and patients with high stress levels and inadequate social support should be given special consideration when designing cardiac rehabilitation programs [42].

Regarding LVEF, the number of grafts, cardiopulmonary bypass time, and cross-clamp time showed no statistically significant impact on all SF-36 subscales. This result aligns with previous studies that showed no direct effect of cardiopulmonary bypass and cross-clamp time on HRQoL after CABG surgery [13, 17]. Additionally, a prospective randomized trial revealed that patients who underwent off-pump CABG without cardiopulmonary bypass showed no cognitive deficits or greater improvement in QoL [42]. In contrast, prolonged cumulative cardiopulmonary bypass time was influential in predicting mortality, postoperative complications, a long-lasting ICU stay, and prolonged mechanical ventilation [44]. Therefore, further confirmation of the safe limits for cardiopulmonary bypass and cross-clamp time might provide new insights for enhancing the intraoperative and postoperative outcomes of patients undergoing CABG [45]. Furthermore, prospective studies are required to elucidate the effect of operation factors, such as the number and type of graft, cardiopulmonary bypass time, and cross-clamp time, on HRQoL after CABG surgery. Additionally, a method of operating, such as off-pump, minimally invasive, and robotic CABG surgery, is needed. In addition, studies should assess the QoL following data collection prospectively to reduce the implications that come with a retrospective review of records, which may lead to bias in the data.

Limitations

This study had some limitations. First, the study had a retrospective cross-sectional design with a small sample size. Consequently, by definition, it did not assess preoperative (baseline) HRQoL, which has always been the main factor influencing postoperative HRQoL. Assessing the quality of life in patients preoperatively will provide us with the data to assess the changes in the quality of life that a patient may experience prospectively following CABG. Therefore, a longitudinal study with a larger sample size, which will provide us with more information and will accurately estimate the mean of our population, and continuous follow-up comparing pre- and postoperative HRQoL and its relationship with other factors would be more beneficial; however, this study could identify factors that affected the QoL of these patients. Second, several factors may have contributed to the results of this study, including inadequately structured cardiac rehabilitation and lifestyle modification programs and awareness regarding the modifiable risk factors of CVDs. In addition, selection bias may have influenced the internal validity of our study, which could have given us inaccurate results regarding the relationship between our variables. These inaccurate estimations affect the external validity as the results become ungeneralizable to the population. Methodologically, this might be interpreted as selection bias, and the results might differ at other medical facilities. Therefore, an unanswered question is whether the preoperative health status should be optimized to be approved to undergo cardiac surgery and to expect a good long-term outcome. Certainly, it is a crucial clinical issue to refer the “right” patients for surgery. Third, the lack of significant postoperative outcomes, such as the causes and incidents of post-CABG myocardial ischemia, can reflect on QoL. This will aid in identifying the group of patients at risk of experiencing suboptimal HRQoL postoperatively, who can then be targeted by support programs of behavioral therapy and psychotherapy pre-operatively and perhaps estimate HRQoL outcomes after CABG surgery. Despite the prognostic relevance of the Charlson Comorbidity Index and the CHA2DS-Vasc (short for congestive heart failure, hypertension, age, diabetes mellitus, stroke, vascular disease, age, sex) Score in the cardiological setting, these scores were not specifically tested in the present study [46, 47].

## Conclusions

Post-CABG patients with congestive heart failure are more likely to have poor general health, vitality, mental health, and limitations due to physical problems. Additionally, patients who experienced PVD also showed a significant negative impact on general health perception. Postoperative complications are considered to be contributing risk factors and have negative effects on both physical functioning and vitality among patients undergoing CABG. Furthermore, lower general and mental health scores were strongly associated with unemployment and widowed status, respectively. Therefore, we believe that identifying and selecting patients who adhere to cardiac rehabilitation programs and follow-up after discharge could improve the QoL of patients undergoing primary isolated CABG by including well-structured and comprehensive care plans, patient education programs, and support services to address the identified risk factors, including congestive heart failure and PVD. The application of additional clinical sessions that are addressed after discharge and are necessary for the management of post-CABG patients needs to be assessed formally and include the consistent and continuous measurement of health conditions to monitor patient recovery for at least six months. Further use of health status instruments is recommended to explore more options and assessment tools that help clinicians and patients assess HRQoL. Accordingly, larger prospective cohort studies will add significant value to the literature by including multi-assessments and valid tools.
